# Improving data collection, documentation, and workflow in a dementia screening study

**DOI:** 10.5195/jmla.2017.221

**Published:** 2017-04

**Authors:** Kevin B. Read, Fred Willie Zametkin LaPolla, Magdalena I. Tolea, James E. Galvin, Alisa Surkis

## Abstract

**Background:**

A clinical study team performing three multicultural dementia screening studies identified the need to improve data management practices and facilitate data sharing. A collaboration was initiated with librarians as part of the National Library of Medicine (NLM) informationist supplement program. The librarians identified areas for improvement in the studies’ data collection, entry, and processing workflows.

**Case Presentation:**

The librarians’ role in this project was to meet needs expressed by the study team around improving data collection and processing workflows to increase study efficiency and ensure data quality. The librarians addressed the data collection, entry, and processing weaknesses through standardizing and renaming variables, creating an electronic data capture system using REDCap, and developing well-documented, reproducible data processing workflows.

**Conclusions:**

NLM informationist supplements provide librarians with valuable experience in collaborating with study teams to address their data needs. For this project, the librarians gained skills in project management, REDCap, and understanding of the challenges and specifics of a clinical research study. However, the time and effort required to provide targeted and intensive support for one study team was not scalable to the library’s broader user community.

## BACKGROUND

Research data management has emerged as a prominent service that librarians offer. Over the past five years, an increasing body of evidence has shown that librarians actively engage in supporting data management plans [1], teaching research data management [2, 3], and curating data [4]. Medical librarians specifically offer a variety of research data management services, including providing instruction, supporting research labs, developing institutional partnerships around data, creating infrastructure, and implementing data science and visualization services [5].

One way that medical librarians have been able to gain exposure to research data management is through the National Library of Medicine (NLM) informationist program. The informationist program was established in 2010 for the purposes of supporting the integration of information professionals into biomedical research teams to develop research data management skills [6]. These supplements have included projects supporting research data management training [7], database creation [8], and research workflow development [9]. The New York University (NYU) Health Sciences Library received a supplement in 2014 for a project to improve the data collection, management, and workflows of a National Institutes of Health (NIH)–funded Research Project Grant (R01) multicultural dementia screening study [10].

The specific aims of the dementia screening study include screening Caucasian, African American, and Hispanic older adults for dementia. In addition to the ongoing R01, the principal investigator (PI) is simultaneously conducting 2 additional multicultural dementia studies with collection of overlapping data. The study involves collecting data in the form of a screening test, medical assessment, and biomarkers from 477 study participants, totaling 643,950 data points. The screenings include both preexisting and PI-developed tests to accurately detect cognitive impairment. Tests that the PI developed include a Lewy body dementia assessment [11] and Quick Dementia Rating System (QDRS) [12], among others. The data collected from all 3 studies will be merged into a large dataset, which the PI intends to share with the broader scientific community at the conclusion of the studies. This case study addresses how 3 librarians collaborated with the study team to improve data collection and organization processes.

## STUDY PURPOSE

**Figure 1:**
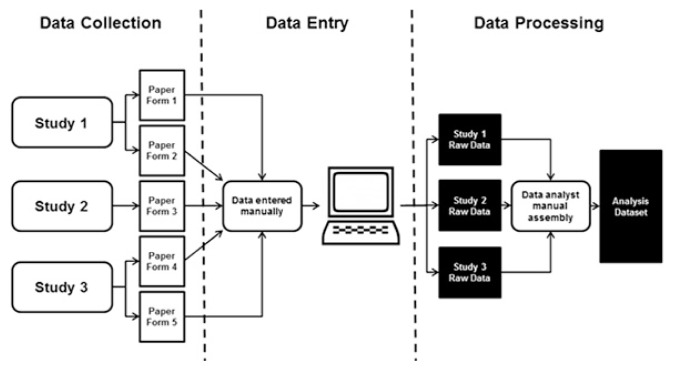
Original study workflow

The librarians’ role in this project was to meet needs expressed by the study team around improving data collection and processing workflows to improve study efficiency and data quality. Before the librarians became involved in this project, the data from the R01-funded study and the 2 other studies conducted by the PI were merged, resulting in the collection of over 900 variables on paper forms. A number of these variables were collected for more than 1 of the 3 studies. The data from those forms were then entered into SPSS statistical software by a data analyst. The data in the forms were represented by variables with unclear, nonstandard names that were not documented in a data dictionary. A data dictionary serves as a guide to the data and includes a descriptive list of names, definitions, and specific values (e.g., male/female/other) for the collected data [13]. Several variables were also identified as compound variables, meaning that 2 data points were collected for a single variable (e.g., drug name and dose). Once entered into SPSS, the process for merging the 3 datasets into 1 analysis dataset increased the risk of error and was not easily reproducible. The workflow described above is depicted in Figure 1. The goal of the librarians was to address challenges concerning the paper form data, large number of unclear variables, and dataset workflow to improve the study team’s overall processes.

## CASE PRESENTATION

The librarians’ involvement in this project focused on improving three domains: data documentation, data collection, and the data processing workflow. A substantial improvement to the efficiency and data quality of the project was achieved through the introduction of an electronic data capture (EDC) system, which is a way to enter data from the study electronically, to replace the use of paper forms. Building the forms in an EDC system involved entering variable names, types (e.g., free text, multiple choice), and when applicable, specific values (e.g., male/female/other). To facilitate building the EDC system, the first task that the librarians undertook involved transforming variable names and creating a data dictionary.

### Data documentation

The variable names that the clinical study team used did not always adhere to available standards and were often unclear. Many of the variable names made use of acronyms and numbers. For instance, the Lewy body dementia screening test listed its variables as LBCRS1, LBCRS2, to LBCRS10, making use of a numbering system that did not reflect the content of each variable. This naming convention made it difficult to understand the data points that were collected in a study and increased the likelihood of errors in data analysis. For example, the variable LBCRS1 was used to measure a study participant’s slowness in initiating and maintaining movement, while LBCRS2 was used to measure rigidity in their range of motion—a small difference in the name for two very different variables. The documentation of these variables was also incomplete in scope, an issue exacerbated by the study’s lack of a data dictionary. Without a complete dictionary, the data were less likely to be useful to those with whom the data might be shared. Furthermore, the lack of a data dictionary combined with unclear variable names created the possibility for new study staff to misinterpret the data.

The first task the librarians undertook was to identify compound variables and transform those to independent variables. For example, a single variable was used to collect data on both the drug name and dose amount; this variable was transformed into 2 separate variables for drug name and dose amount. Compound variables increase the possibility of confusion in data collection, error in data entry, and error and difficulty when analyzing the data. After transforming all compound variables into independent variables, the total number of variables increased from over 900 to more than 1,200.

Next, the librarians sought to transform the variable names to adhere to naming conventions from established data standards. Standards provide established frameworks, including naming conventions, that serve to improve the consistency of data collection across all types of research. These frameworks make the data more understandable to those who use the same standard and increase the chances of interoperability because the data have been structured in a standardized way [14]. For this study, the librarians chose to use the Clinical Data Interchange Standards Consortium’s Clinical Data Acquisition and Harmonization (CDASH) standard [15] to modify the study’s variable naming conventions. CDASH provided guidelines and variable naming conventions for eighteen domains including demographics, medical history, and physical examination [16], all of which were included in the PI’s studies. When CDASH did not address specific variables in the study, the librarians used the National Institute of Neurological Disorders and Stroke (NINDS) Common Data Elements (CDEs) [12], a data collection standard used in the PI’s research community.

For variable names that were not found in either CDASH or the NINDS CDEs, the librarians developed naming conventions in consultation with the data analyst. When transforming the PI’s customized screening tests, the librarians transformed cryptic variable names to ones that were more descriptive and, therefore, more comprehensible. For example, variables from the Lewy body dementia assessment like “LBCRS1” were transformed to “LBCRS_slowness” to better represent the meaning of the variable.

Once the variable name transformation process was complete, the librarians prepared the infrastructure for a data dictionary. The librarians provided the study team with a structure for how the data dictionary should be developed and solicited information from them to document the transformed variable set. The structure of the data dictionary included: the variable name, the field label name that represented the written instructions on the clinical form, the attributes for each variable (e.g., for gender, 1=male, 2=female, and 3=other), the calculations for each variable (e.g., body mass index=kg/m2), validation restrictions (e.g., age between 18 and 65), and whether the variable included protected health information.

### Data collection

As mentioned previously, the PI has 3 studies that collect multicultural dementia screening data from study participants. One of the studies had a cross-sectional design using a single form for data collection, while the other 2 studies were longitudinal and used both an initial form and a follow-up form, for a total of 5 data collection forms. At the studies’ outset, over 900 variables were being collected across all forms. Data were collected at individual sites using paper forms, with different study administrators managing data collection for each study. Those paper forms were then sent to the study team’s data analyst, who manually entered the data into SPSS statistical software (Figure 1). Opportunities for error increased with the complexity of 900 variables being collected on 5 separate paper forms across 3 studies, which in turn were manually entered by a single data analyst. This manual entry also took a significant amount of time, which delayed analysis.

**Figure 2:**
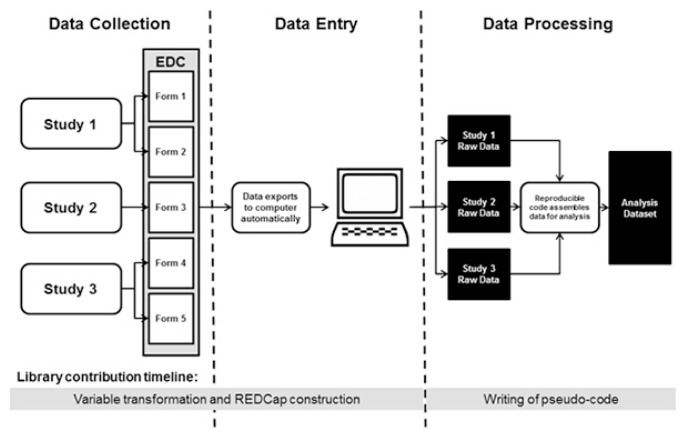
Revised study workflow after completion of informationist project

To improve data quality and study efficiency, the librarians proposed to recreate the 5 forms—now comprising over 1,200 variables following the variable transformation—in the REDCap EDC system [17]. REDCap is a widely available, easy-to-learn tool that supports electronic data collection. It was supported by the authors’ academic medical center and provided many features that addressed the study team’s needs. REDCap can help eliminate data entry errors because its data validation feature allows specification of whether a variable should be a certain type (e.g., a specified date format) or be within a certain range, so that anyone entering data that do not match the specification will be alerted. REDCap also supports detailed user permission controls so that study personnel can be limited in how they interact with the data. For statistical analyses, data stored in REDCap can be exported into a variety of statistical software formats. This feature improved the study team’s efficiency when entering the data into SPSS by reducing the process to an export, rather than spending many hours completing error-prone manual data entry (Figure 2). Additionally, REDCap is Health Insurance Portability and Accountability Act (HIPAA)–compliant and allows each variable to be flagged if it contains protected health information, allowing later export of a de-identified dataset that aligns with the PI’s goal of sharing the data.

Another feature of REDCap is the bulk upload of form elements. REDCap allows form creation via an online designer or by bulk uploading of a formatted spreadsheet (in CSV format). In creating 5 forms that encompassed over 1,200 variables, the bulk upload feature was invaluable to the librarians’ effort and resulted in a time savings of many hours. Even with the bulk upload feature available and a data dictionary built in SPSS (allowing easy copying and pasting to the REDCap-formatted CSV), the process of building the REDCap forms took several weeks’ worth of full-time effort involving repeated communication with the study team to get the forms in a satisfactory condition. Calculated fields were a particular challenge, as these required working within REDCap’s syntax, which can quickly become complex when adding multiple fields.

### Data processing workflow

The final area that the librarians addressed was the workflow used to create the datasets used for analysis. The raw data consisted of the three separate datasets, one from each study. The raw datasets were then combined by the data analyst into one analysis dataset. This process was done manually in SPSS, rather than programmatically (Figure 1). A complicating factor in combining the datasets was that some variables were collected for more than one study, and some subjects were enrolled in more than one study. This resulted, in some cases, in variables that were collected multiple times (once for each study) for the same subject. For those variables, the data analyst would compare the date of collection and only include the most recently collected data point in the analysis dataset. This process of manually extracting variables subject by subject from the raw datasets was more likely to produce errors than a script that could create analysis datasets that are reproducible and easily comprehensible to others.

The librarians worked with the data analyst to outline the workflow used to construct the analysis dataset. Once the workflow was described, one of the librarian authors who had an extensive coding background provided the data analyst with pseudocode to perform the process. Pseudocode is a series of steps written in natural language, rather than in any particular coding language, that outlines the logic that would need to be written into code. The pseudocode comprehensively described the logic needed to reproducibly construct the analysis dataset. The division of responsibilities outlined at the beginning of the project left the actual implementation of the code to the data analyst.

The introduction of the REDCap database and the new workflow created a streamlined and reproducible path from data collection to data analysis (Figure 2). All of the data (including the data dictionary) is stored securely in REDCap behind the institutional firewall, and the analyst can export datasets for use when necessary. Once the pseudocode is implemented by the data analyst, it can then be run on the raw datasets to create a new analysis dataset, rather than continuing to use a manual process to add to an existing analysis dataset.

At the time of writing this paper, the REDCap database with new variables names has been fully developed, and the study team responsible for collecting data has been trained on using REDCap by one of the librarian authors. The data analyst is now in the process of uploading all previously collected data (from the paper forms) into the REDCap database. Once this process is complete, the study team will use the REDCap database exclusively to collect data for the ongoing R01 and two other dementia screening studies, saving the study team a significant amount of time and effort with data entry. Concerning the data processing workflows, the data analyst is still in the process of using the pseudocode to develop actual code in SPSS to create new analysis datasets that can then be shared with the research community.

## DISCUSSION

The NLM informationist projects are invaluable for providing librarians with an opportunity to work with researchers and develop research data management skills. While the librarians on this project benefitted from having some background in research data management and coding, this project provided us with new skills in understanding research workflows and using REDCap. The experience gained collaborating with researchers has provided the librarians with a stronger understanding of the research process, which has improved interactions with our user community when discussing their data management needs.

Collaborating with a study team to improve data management practices was an enlightening experience for both the librarians and study team. The study team, while deeply knowledgeable about their study and subject matter, was less familiar with the processes and workflows for managing and combining multiple datasets. Conversely, the librarians’ experience organizing and managing information aligned well with the specific aims of this project. To gain buy-in from study personnel, the librarians provided specific examples of how and why each proposed solution would benefit the study. Librarians participating in informationist projects should be prepared to demonstrate the value that they will bring to the project throughout the process.

It was also critical to outline the distribution of labor before the project began. From the outset, the librarians developed clear project goals, set expectations for project outcomes, outlined the specific tasks the librarians were responsible for, and specified all the tasks required of the study team. These last two steps were crucial when the librarians transformed the variables to a new naming convention. The project documentation specified that the librarians would complete the mapping between the old and newly renamed variables, while the data analyst would be responsible for transforming the variables for the existing data and uploading that data into the new REDCap database. We highly encourage librarians who are looking to begin an NLM informationist project or other large-scale collaboration to consider this approach, as documentation can serve as a resource for holding a study team accountable for project tasks.

When taking on this type of collaboration, it is also important to consider the scalability of the provided services. While the librarians on this project gained valuable experience and skills using REDCap, navigating the research process, and creating pseudocode, the level of effort required to provide these services far exceeded the allocated time and budget requirements offered by the informationist supplement. Furthermore, it was not feasible for the librarians to offer the same level of service that was provided in the informationist project to other individuals in our medical center user community. When considering committing to data management projects of this scale, it is important to weigh the amount of time that will be needed to complete the project against the value of potential outcomes for the librarians and their user communities. Determining this balance can be challenging, but we encourage librarians to be open minded about data management opportunities—including those that might not be scalable—while considering what aspects of these opportunities can lead to scalable services. For example, the skills that the librarians developed in this project initiated scalable opportunities to regularly teach REDCap and provide consultations to our faculty and students. These opportunities would not have been possible without our participation in the informationist project.

NLM informationist projects highlight the “added value” that librarians can bring to medical research when they support research data management as part of a parent grant application and provide opportunities for developing new scalable data management services. We encourage librarians interested in gaining experience and skills in research data management to consider applying for future informationist supplements.
